# The Hospital Penny Fund

**Published:** 1904-11-12

**Authors:** 


### "THE HOSPITAL PENNY FUND."

We are informed that the promoters have convened a meeting at the Blenheim Rooms, Hôtel Cecil, on Tuesday next, 15th inst., at 2.30. The papers and proposals submitted do not make the scheme less objectionable, although the promoters' representatives who have called on certain hospital secretaries have made great use of Mr. George Herring's name. Mr. George Herring is one of the most retiring of men, and we have little doubt that he will be exceedingly annoyed, as he has good reason to be, by the proceedings in question. The two representatives of the Penny Fund, Messrs. Stokes and Warren, informed one of the principal hospital secretaries in London that all the hospital representatives would attend the meeting on Tuesday with the object of reorganising the Hospital Penny Fund. When, however, the notice came four days afterwards, with enclosures, he could not discover the name of any such representatives. On inquiry, too, the hospital secretary referred to ascertained that the hospital authorities are still holding aloof from the Fund, and had no intention of attending the meeting on Tuesday.

However, it may be realised during the next few days that the shortest way to put an end to the matter would be for the hospital secretaries to attend the meeting and to insist upon the whole scheme being abandoned. Mr. John Murray has well pointed out that no one has a right to use the name of a hospital without the consent of those responsible for the management of its affairs. The hospitals have therefore the matter entirely in their own hands, and should act together and decisively.

### What of the Prizes and Results?

In *Public Opinion* the promoters under the head of the "Main Point to remember," stated that "Our Prize Scheme will be as follows :"—"Every month for the next four months we shall give some 18 cash prizes, aggregating £400, and 134 consolation prizes valued at 10s. 6d. each."

*Public Opinion* of November 4th publishes the prize-winners, the prizes offered, and the stamps bought. If the prizes offered have been awarded, as the statement in *Public Opinion* seems to show, the financial results of the first month are as follows :—

The prizes were offered in four groups. In Group 1 the actual number of stamps bought was £52 19s. The first prize-winner bought £20 of stamps, and received presumably £200 in cash. The second bought £15 10s. of stamps, and received £50 in cash.

In the second group the prizes amount to £75. Here a purchaser bought 25s. worth of stamps and received £50. An investment of 20s. brought the second competitor £20 in cash, and for 15s. spent on stamps the third received £5.

In the third group a boy who spent 10s. in stamps received £5 ; 7s. 6d. brought another boy £2 in cash, and 3s. brought a third boy £1 in cash.

Group 4.—Here the girls were not so business-like. The first prize-winner laid out 25s. in stamps and received £5, whilst the second invested 2s. and received £2.

In the result we gather from *Public Opinion* that for an expenditure in cash of £346 in prizes the promoters have managed to dispose of stamps to the value of £50 17s. 6d.

Of the 134 consolation prizes offered 22 appear to have been awarded, of which one-half were given to persons who laid out one shilling each in stamps. Hence an outlay of £11 11s. 0d. in prizes yielded twenty-two shillings from the sale of stamps. In face of such a fiasco as this it is not a little remarkable that the promoters should have had the hardihood to summon a meeting at all, much less with the object of continuing the work of a Fund like this. The fact is that, quite apart from the merits of this or any other scheme for raising money by the sale of stamps, the experience of the King's Fund proves that the public will not buy labels, and the promoters are only offering labels for sale. It does not matter under whose auspices such a Fund might be promoted, for any system of selling labels is bound to fail. If a distribution of £346 in cash has realised less than £60 by the sale of these labels, mis-called stamps, it is demonstrably proved that the scheme as it stands is a patent folly, which ought never to have been attempted, and should now be buried in oblivion as speedily as possible.

We might say a great deal more but in view of the fiasco which has resulted from this attempt to exploit the hospitals and to sell labels to the public under the name of stamps, we defer further comment until after the meeting on Tuesday next.

We hope, however, that the daily papers will send reporters to the meeting on Tuesday, and that the promoters will make proper provision for the accommodation of the Press. Heretofore the proceedings of this "Great Hospital Scheme" have been conducted with a privacy which has not proved to the public advantage.

